# Impact of the Dopamine System on Long‐Term Cognitive Impairment in Parkinson Disease: An Exploratory Study

**DOI:** 10.1002/mdc3.13751

**Published:** 2023-04-25

**Authors:** Daniel Weintraub, Marina Picillo, Hyunkeun Ryan Cho, Chelsea Caspell‐Garcia, Cornelis Blauwendraat, Ethan G. Brown, Lana M. Chahine, Christopher S. Coffey, Roseanne D. Dobkin, Tatiana Foroud, Doug Galasko, Karl Kieburtz, Kenneth Marek, Kalpana Merchant, Brit Mollenhauer, Kathleen L. Poston, Tanya Simuni, Andrew Siderowf, Andrew Singleton, John Seibyl, Caroline M. Tanner, the Parkinson's Progression Markers Initiative

**Affiliations:** ^1^ Department of Psychiatry Perelman School of Medicine at the University of Pennsylvania Philadelphia Pennsylvania USA; ^2^ Assistant Professor in Neurology at the Department of Medicine, Surgery and Dentistry “Scuola Medica Salernitana” University of Salerno Italy; ^3^ Department of Biostatistics, College of Public Health University of Iowa Iowa City Iowa USA; ^4^ Center for Alzheimer's and Related Dementias, and the Integrative Neurogenomics Unit, Laboratory of Neurogenetics National Institute on Aging, National Institutes of Health Bethesda Maryland USA; ^5^ Department of Neurology Weill Institute for Neurosciences, University of California, San Francisco San Francisco California USA; ^6^ Department of Neurology University of Pittsburgh Pittsburgh Pennsylvania USA; ^7^ Department of Psychiatry Rutgers University, Robert Wood Johnson Medical School Piscataway New Jersey USA; ^8^ Department of Medical and Molecular Genetics Indiana University Indianapolis Indiana USA; ^9^ Department of Neurology University of California San Diego California USA; ^10^ Department of Neurology University of Rochester Medical Center Rochester New York USA; ^11^ Institute for Neurodegenerative Disorders New Haven Connecticut USA; ^12^ Department of Neurology Northwestern University Feinberg School of Medicine Chicago Illinois USA; ^13^ Department of Neurology University Medical Center Goettingen Goettingen Germany; ^14^ Department of Neurology and Neurological Sciences Stanford University Stanford California USA; ^15^ Department of Neurology Perelman School of Medicine, University of Pennsylvania Philadelphia Pennsylvania USA; ^16^ Center for Alzheimer's and Related Dementias, and the Molecular Genetics Section Laboratory of Neurogenetics, National Institute on Aging, National Institutes of Health Bethesda Maryland USA

**Keywords:** dopamine, cognition, Parkinson's disease

## Abstract

**Background:**

Little is known about the impact of the dopamine system on development of cognitive impairment (CI) in Parkinson disease (PD).

**Objectives:**

We used data from a multi‐site, international, prospective cohort study to explore the impact of dopamine system‐related biomarkers on CI in PD.

**Methods:**

PD participants were assessed annually from disease onset out to 7 years, and CI determined by applying cut‐offs to four measures: (1) Montreal Cognitive Assessment; (2) detailed neuropsychological test battery; (3) Movement Disorder Society‐Unified Parkinson's Disease Rating Scale (MDS‐UPDRS) cognition score; and (4) site investigator diagnosis of CI (mild cognitive impairment or dementia). The dopamine system was assessed by serial Iodine‐123 Ioflupane dopamine transporter (DAT) imaging, genotyping, and levodopa equivalent daily dose (LEDD) recorded at each assessment. Multivariate longitudinal analyses, with adjustment for multiple comparisons, determined the association between dopamine system‐related biomarkers and CI, including persistent impairment.

**Results:**

Demographic and clinical variables associated with CI were higher age, male sex, lower education, non‐White race, higher depression and anxiety scores and higher MDS‐UPDRS motor score. For the dopamine system, lower baseline mean striatum dopamine transporter values (*P* range 0.003–0.005) and higher LEDD over time (*P* range <0.001–0.01) were significantly associated with increased risk for CI.

**Conclusions:**

Our results provide preliminary evidence that alterations in the dopamine system predict development of clinically‐relevant, cognitive impairment in Parkinson's disease. If replicated and determined to be causative, they demonstrate that the dopamine system is instrumental to cognitive health status throughout the disease course.

**TRIAL REGISTRATION:**

Parkinson's Progression Markers Initiative is registered with ClinicalTrials.gov (NCT01141023).

Cognitive impairment (CI) is among the most problematic outcomes in Parkinson disease (PD). Prospective, longitudinal studies have found that dementia (PDD) occurs in up to 80% of patients.^1^ Additionally, 25% to 30% of non‐demented patients have mild cognitive impairment (PD‐MCI),^2^ 10–20% of newly‐diagnosed patients have cognitive deficits or early decline after diagnosis,^3^ and changes in cognition have even been reported prior to diagnosis.^4^, ^5^

In spite of the high prevalence and clinical importance of CI in PD, there are few treatment options. Current pharmacological interventions for PDD are limited to those developed for Alzheimer's disease (AD), with modest treatment benefit for cholinesterase inhibitors and no clear benefit for memantine,^6^ and treatment studies for PD‐MCI have not been promising.^6^

Strides in understanding the pathophysiological changes of CI in PD have been made recently. Pathology studies have demonstrated that diffuse Lewy bodies appear to be the major contributing pathology to decline,^7^ with up to one‐third of PDD patients also having AD‐related changes.^8^ Neurotransmitter deficits linked with CI include acetylcholine, dopamine, and norepinephrine.^9^ It is likely that pathological and neurochemical heterogeneity underpins cognitive decline in PD, with disruptions to multiple neural networks.

Dysfunction of the dopamine system (DS) is central to the diagnosis and progression of PD, particularly its motor features. However, relatively little is known about how the DS impacts the cognitive course of the disease. It has been hypothesized that early CI in PD is driven by dopaminergic deficits, and that later, more severe deficits are due to cortical cholinergic dysfunction.^10^, ^11^ Deficits in the DS, assessed primarily with dopamine transporter (DAT) SPECT imaging, have been associated with decreased global^12^, ^13^ and specific^14^, ^15^, ^16^ cognitive abilities in preliminary studies, including in early PD.^17^, ^18^, ^19^

Genetic predictors of long‐term cognitive decline in PD are apolipoprotein ε4 (*APOE* ε4) allele and glucocerebrosidase (*GBA*) mutations.^20^ Regarding dopamine‐related single‐nucleotide polymorphisms (SNPs), the *COMT val* ^158^ *met* genotype has been associated with PD‐MCI,^21^, ^22^ and the *DRD2C957T* genotype correlated with increased risk for PDD.^21^ Another, cross‐sectional study reported associations with two *DRD2* SNPs and dementia.^23^

Research has demonstrated that levodopa may have acute or short‐term beneficial^24^, ^25^ or detrimental^26^, ^27^ effects on cognitive performance; however, there is no evidence initial choice of dopamine replacement therapy (DRT) makes a difference in terms of subsequent dementia,^28^, ^29^ and a trial of a selective monoamine oxidase B inhibitor was negative for treatment of PD‐MCI.^30^

Few studies have examined a large cohort of PD patients from disease onset annually for up to 7 years focused on development of defined CI. The impact of three aspects of the DS on cognition have not been evaluated in a single study: (1) DAT integrity; (2) dopamine‐related SNPs; and (3) total dopaminergic medication exposure. Results would help inform whether longitudinal CI in PD has dopaminergic contributions, and if disease‐modifying or novel symptomatic therapies targeting the DS might be beneficial.

## Methods

### Standard Protocol Approvals, Registrations, and Patient Consents

An ethical standards committee on human experimentation reviewed and approved the study at each site. Additionally, the Western Institutional Review Board at the University of Florida reviewed this study (Protocol #20200597). At the University of Pennsylvania, the Institutional Review Board approved this research (Protocol #843441). All participant signed an approved informed consent form. We confirm that we have read the Journal's position on issues involved in ethical publication and affirm that this work is consistent with those guidelines.

### Participants

Study methodology have been published.^31^, ^32^ Parkinson's Progression Markers Initiative (PPMI) recruited individuals with newly‐diagnosed, untreated PD (i.e., duration ≤2 years) at baseline and not expected to require DRT for at least 6 months. Reduced DAT SPECT binding was required for inclusion, and those with dementia were excluded.

### Clinical Variables

Clinical variables were examined as possible co‐variates if associated with CI in previous research. Fixed variables were age at enrollment, sex, education level and race. Time‐varying variables for longitudinal analyses were REM Sleep Behavior Disorder Screening Questionnaire (RBDSQ)^33^ score, total State–Trait Anxiety Inventory (STAI)^34^ score, 15‐item Geriatric Depression Scale (GDS‐15)^35^ score, Movement Disorder Society‐Unified Parkinson's Disease Rating Scale (MDS‐UPDRS)^36^ motor (Part III) score (“off” score at baseline and “on” score at subsequent visits), Anticholinergic Cognitive Burden (ACB) scale^37^ score (assessment of anticholinergic burden), and cognitive‐enhancing medication use ie, either acetylcholinesterase inhibitors or memantine.

### Definition of Cognitive Impairment

To maximize sensitivity to detect a neurobiological signal, and given that a variety of ways are used to define CI in PD, four distinct definitions of CI were utilized: (1) *MoCA*: score < 26, as previously recommended^38^; (2) *detailed cognitive testing*: ≥2 tests impaired (>1.5 below standardized mean) from a cognitive battery of five tests (memory: Hopkins Verbal Learning Test—Revised HVLT‐R, using both immediate free recall and recognition discrimination scores); visuospatial function: Benton Judgment of Line Orientation 15‐item version; processing speed‐attention: Symbol‐Digit Modalities Test; and executive function and working memory: Letter‐Number Sequencing and semantic (animal) fluency), as previously defined^39^; (3) *MDS‐UPDRS cognition* (Part I) score ≥2 (at least “mild” CI; and^4^ *investigator diagnosis* (site investigator diagnosis of either PD‐MCI^40^ or PD dementia,^41^ guided by consensus criteria that consider subjective cognitive change, objective cognitive performance, and day‐to‐day cognitive functioning). The investigator cognitive categorization is a partial dataset (data for 104/417 participants at baseline and 269/391 participants at year 1).

CI was determined at baseline and at annual visits for the four impairment measures. The longitudinal characterization of CI was done two ways: (1) including all participants and considering CI at each visit separately for each measure, including baseline impairment on the selected test (“*impairment*”); and (2) including a subgroup of participants who developed incident, persistent CI on a given cognitive measure versus those participants who were never impaired on that measure (“*persistent impairment*”). For the latter categorization, the impaired group could not be impaired at baseline, were required to have at least one visit after conversion, and once converted had to stay converted at future visits. The unimpaired could never be impaired at any visit for the selected measure and were required to have at least one post‐baseline assessment. All other participants were excluded from the persistent CI analysis.

### Dopamine System Variables

PPMI methodology for biological variable collection and analyses have been reported^31^, ^32^ (https://www.ppmi-info.org/study-desing/research-documents-and-sop), as has calculation of levodopa equivalent daily dose (LEDD).^42^

#### DaTscan SPECT

Two values for striatal dopamine integrity (DAT SPECT) based on DaTscan results were used: (1) mean specific binding ratio (SBR) value (the average of right caudate, left caudate, right putamen and left putamen) and (2) lowest putaminal SBR. Both measures are corrected for the normal loss of DATs associated with aging. The mean value provides a good summary assessment with robust counting statistics, while the lowest putamen is more a severity index, although suffers from more statistical noise. DaTscan values for baseline and years 1 to 5 were used for longitudinal models. Four hundred thirteen participants had a DaTscan at baseline, 367 at year 1, 357 at year 2 or 3, and 299 at year 4 or 5.

#### Genetics

Genetic data were obtained from https://www.ppmi-info.org/. Single nucleotide polymorphisms (SNPs) previously associated with the DS were included (Table 1). A SNP was removed if it was in high linkage disequilibrium (*r* ^2^ > 0.8) with another SNP, resulting in the removal of DRD1 rs4532, DRD1 rs265981, SLC18A2 rs363224, and MAO‐B rs6651806. SNPs were analyzed as dichotomous variable (presence of one or two copies of Allele 2).

**TABLE 1:** Participant characteristics longitudinally

Variable	Baseline (N = 417)	Year 1 (N = 391)	Year 2 (N = 375)	Year 3 (N = 363)	Year 4 (N = 344)	Year 5 (N = 314)	Year 6 (N = 273)	Year 7 (N = 239)
Cognitive Impairment								
MoCA, %	21.6	34.5	32.4	31.9	30.1	28.3	32.1	28.2
Detailed cognitive testing, %	15.4	18.5	16.0	18.6	17.5	18.4	17.3	19.8
MDS‐UPDRS cognition score, %	3.1	4.1	8.5	9.4	11.4	12.4	12.6	16.4
Investigator diagnosis, %	7.7	14.5	16.3	21.9	21.7	19.7	21.8	28.5
Dopamine system								
Mean striatum DAT SBR, mean (SD)	1.40 (0.39)	1.24 (0.35)	1.16 (0.37)	0.96 (0.27)	1.02 (0.34)	0.91 (0.47)	NA	NA
Lowest putamen DAT SBR, mean (SD)	0.33 (0.12)	0.29 (0.10)	0.27 (0.11)	0.21 (0.08)	0.24 (0.10)	0.22 (0.18)	NA	NA
LEDD, mean (SD)	N/A	180.95 (231.14)	333.19 (314.89)	444.68 (349.06)	529.68 (352.03)	613.85 (325.05)	709.20 (438.98)	763.63 (463.12)
Covariates								
RBDSQ score, mean (SD)	4.2 (2.7)	4.2 (2.8)	4.6 (3.0)	4.7 (2.9)	5.0 (3.2)	5.0 (3.1)	5.2 (3.2)	5.7 (3.3)
STAI score, mean (SD)	65.3 (18.1)	65.1 (18.7)	65.0 (18.6)	64.8 (18.8)	64.7 (18.8)	64.9 (19.4)	66.2 (19.0)	67.9 (18.9)
GDS‐15 score, mean (SD)	2.3 (2.4)	2.6 (2.9)	2.6 (2.9)	2.6 (2.8)	2.6 (2.8)	2.8 (2.8)	2.8 (2.8)	3.2 (3.1)
MDS‐UPDRS Part III score, mean (SD)	20.9 (8.9)	23.1 (10.8)	22.9 (11.3)	23.9 (12.1)	24.0 (12.8)	24.5 (13.2)	24.1 (12.3)	24.4 (12.2)
ACB score, mean (SD)	0.7 (1.3)	1.1 (1.6)	1.2 (1.8)	1.4 (1.9)	1.4 (1.9)	1.5 (1.9)	1.5 (1.9)	1.7 (2.0)
Cognitive‐enhancing medication use, %	0	0.5	1.3	2.8	4.7	5.1	5.1	6.3

Abbreviations: ACB, anticholinergic cognitive burden; DAT, dopamine transporter; GDS‐15, 15‐item Geriatric Depression Scale; MDS‐UPDRS, Movement Disorder Society‐Unified Parkinson's Disease Rating Scale; MoCA, Montreal Cognitive Assessment; RBDSQ, REM Sleep Behavior Disorder Screening Questionnaire; SBR, striatal binding ratio; STAI, State–Trait Anxiety Inventory.

#### LEDD

Total LEDD was zero at baseline as participants were untreated, and was calculated at post‐baseline visits, including all PD medications.^42^ In addition, separate LEDDs were calculated for levodopa only and dopamine agonists only.

### Analyses

Data were downloaded from Laboratory of Neuroimaging (LONI) on February 1, 2021. Statistical analyses were performed using programming language R 4.2.0. Data out to 7 years were utilized. Association between SNPs and longitudinal CI was assessed using generalized estimating equations (GEEs) under a first‐order autoregressive (AR‐1) correlation structure in generalized linear models (GLM) with the logit link function. To assess the long‐term impact of changes in DaTscan and LEDD on CI a two‐step procedure was implemented: (1) mixed effect analyses were conducted to assess the individual level of changes in DaTscan and LEDD, and (2) GEEs under the AR‐1 were used to assess the associations between the changes in DaTscan and LEDD and CI during follow‐up. Association between LEDD change and DaTscan and MDS‐UPDRS motor score changes over time were assessed using GEEs under the AR‐1 in GLM with an identity link function. Clinical and demographic variables with p‐value <0.3 on univariate analyses were included as covariates in all GLMs. A quadratic function of time was included in univariate and multivariate longitudinal analyses. For analyses of CI, time‐varying covariates and baseline impairment status were included, when applicable, and baseline CI status was also included; for persistent CI baseline values for all covariates were utilized, and cognitive‐enhancing medication use was not included as it was not utilized by anyone at baseline.

To account for multiple comparisons, we applied a family‐wise error rate to each set of analyses, with a set being a specific cognitive outcome for a given biomarker predictor (4 DaTscan variables per cognitive outcome, and 18 SNPs per cognitive outcome). Specifically, this was a Bonferroni‐corrected significance level, computed as 0.05/number of family‐wise hypotheses tested, for each cognitive outcome for DaTscan (Table 4) and SNP (Table 5) predictors.

## Results

### Participant Characteristics and Cognitive Impairment

#### Baseline

Four hundred twenty‐three individuals with PD were enrolled, 417 completed baseline assessments, and 238 participants had year 7 data available (for the latter, *N* = 234 for MoCA, *N* = 232 for detailed cognitive testing, *N* = 238 for MDS‐UPDRS cognition score, and *N* = 235 for investigator diagnosis).

Mean (SD) age was 61.6 (2.4) years, males represented 65.2% of the sample, whites were 92.3%, and mean (SD) education level was 15.6 (3.0) years. See Supplementary Table S1 for SNP frequency. Rates (in descending frequency) of CI at baseline were 21.6% (90/417) for the MoCA, 17.6% (64/416) for detailed cognitive testing, and 3.1% (13/417) for the MDS‐UPDRS cognition score (Table 1).

#### Cognitive Impairment

By year 7 prevalence of CI was highest for investigator diagnosis (28.5%) and using the MoCA cut‐off score (28.1%), and lowest for MDS‐UPDRS cognition score (16.4%) (Table 1). The greatest increase in prevalence from baseline to year 7 was in investigator diagnosis (from 7.7% to 28.5%), and at this time point almost half (48.4%) of participants met criteria for 1 or more of the 4 definitions of CI (1 cognitive outcome = 21.6%, 2 = 14.7%, 3 = 7.8%, and all 4 = 4.3%).

#### Persistent Cognitive Impairment

The cumulative proportion of persistent CI over the 7‐year course of observation were: (1) MoCA cut‐off (N = 36; 18%); (2) detailed cognitive testing (N = 16; 7%); (3) MDS‐UPDRS cognition score ≥2 (N = 23; 8%); and^4^ investigator diagnosis (N = 12; 9%).

### Clinical and Demographic Correlates of Longitudinal Impairment

#### Cognitive Impairment

Predictors of CI were: (1) for MoCA: higher age, male sex, lower education, non‐White race, higher STAI, and higher GDS‐15 and higher MDS‐UPDRS motor scores; (2) for detailed cognitive testing: male sex, non‐White race, and higher STAI and GDS‐15 scores; (3) for MDS‐UPDRS cognition score: increasing age, male sex, higher STAI, GDS‐15, and MDS‐UPDRS motor scores; and for (4) investigator diagnosis: increasing age, male sex, lower education, non‐White race, and higher STAI and MDS‐UPDRS motor scores (Table 2).

**TABLE 2:** Demographic and clinical correlates of longitudinal cognitive impairment

Variable	Cognitive impairment outcome			
	MoCA estimate (*P* value)	Detailed cognitive testing estimate (*P* value)	MDS‐UPDRS cognition score estimate (*P* value)	Investigator diagnosis estimate (*P* value)
Cognitive impairment^a^				
Age	0.078 (<0.001)	–	0.0534 (<0.001)	0.080 (<0.001)
Sex	0.592 (0.003)	0.624 (0.005)	0.570 (0.02)	0.479 (0.03)
Education	−0.103 (<0.001)	–	–	−0.104 (0.001)
Ethnicity	−1.107 (0.001)	−0.963 (0.004)	–	−0.916 (0.01)
RBDSQ score	–	–	0.109 (<0.001)	–
STAI score	0.009 (0.005)	0.010 (0.008)	0.029 (<0.001)	0.012 (0.004)
GDS‐15 score	0.064 (<0.001)	0.063 (0.001)	0.219 (<0.001)	–
MDS‐UPDRS motor score	0.014 (0.005)	–	0.035 (<0.001)	0.013 (0.03)
Persistent cognitive impairment^b^				
Age	0.144 (<0.001)	0.102 (0.004)	0.073e (0.004)	0.074 (0.04)
Sex	–	1.492 (0.05)	–	–
Education	–	–	–	–
Ethnicity	−1.636 (0.007)	–	–	–
RBDSQ score	0.237 (<0.001)	–	0.202 (0.007)	–
STAI score	–	–	–	–
GDS‐15 score	–	–	–	–
MDS‐UPDRS motor score	0.071 (0.002)	–	–	–
ACB score	–	–	–	0.538 (0.01)

^a^ Baseline or time‐varying values for predictors of cognitive impairment.

^b^ Baseline values were used as predictors of cognitive impairment.

#### Persistent Cognitive Impairment

Predictors of impairment were: (1) for MoCA: higher age, non‐white race, and higher RBDSQ and MDS‐UPDRS motor scores; (2) for detailed cognitive testing: higher age and male sex; (3) for MDS‐UPDRS cognition score: increasing age and higher RBDSQ score; and (4) for investigator diagnosis: increasing age and higher ACB score (Table 2).

### Dopamine System Predictors of Cognitive Impairment

#### DAT Scan Models

For CI, lower baseline mean striatum SBR DAT was associated with worse detailed cognitive testing over time (*P* = 0.005) (Table 3). For persistent CI, lower baseline mean striatum was associated with worse detailed cognitive testing (*P* = 0.003) and MDS‐UPDRS cognition score over time (*P* = 0.02), but the latter score was not significant after correction for multiple comparisons.

**TABLE 3:** Dopamine transporter SBR and cognitive impairment

Cognitive outcome	Brain region			
	Baseline mean striatum SBR estimate (*P* value)	Baseline lowest putamen SBR estimate (*P* value)	Change over time in mean striatum SBR estimate (*P* value)	Change over time in lowest putamen SBR estimate (*P* value)
Cognitive impairment				
MoCA	−0.038 (0.87)	−0.223 (0.76)	−3.224 (0.38)	−7.450 (0.59)
Detailed cognitive testing	−0.723 (0.005)*	−1.109 (0.16)	3.007 (0.45)	21.124 (0.18)
MDS‐UPDRS cognition score	−0.502 (0.10)	−1.037 (0.36)	−3.072 (0.53)	6.929 (0.71)
Investigator diagnosis	0.126 (0.61)	−0.195 (0.82)	−5.845 (0.13)	−9.804 (0.52)
Persistent cognitive impairment				
MoCA	−0.547 (0.41)	0.418 (0.83)	7.333 (0.43)	0.128 (0.99)
Detailed cognitive testing	−3.201 (0.003)*	−4.652 (0.11)	25.880 (0.12)	31.880 (0.41)
MDS‐UPDRS cognition score	−1.627 (0.02)	−3.880 (0.08)	−4.679 (0.65)	22.109 (0.54)
Investigator diagnosis	−1.640 (0.07)	−6.361 (0.08)	1.106 (0.91)	14.906 (0.74)

* Significant after Bonferroni correction (*P* value <0.0125).

Given the association between the caudate specifically and cognitive abilities in PD,^18^, ^43^ we subsequently examined the association between caudate DAT binding and cognitive outcomes. Lower baseline caudate DAT SBR predicted CI based on cognitive test score (*P* = 0.003) and persistent CI based on cognitive test score (*P* = 0.004) and MDS‐UPDRS cognition score (*P* = 0.03). Lower longitudinal caudate DAT binding predicted persistent CI based on MDS‐UPDRS cognition score (*P* = 0.05).

#### Genetic Models

For CI MAO‐B rs5905512 (A allele) was associated with MoCA impairment (*P* = 0.03), and SLC18A2 rs363387 (T allele) was associated with MDS‐UPDRS cognition score (*P* = 0.01) (Table 4). For persistent CI, DRD3 rs6280 (C allele) associated with MoCA score (*P* = 0.02); DRD4 rs1800955 (C allele) predicted MDS‐UPDRS Part I cognition score (*P* = 0.05); and both SLC18A2 rs363276 (T allele) and SLC18A2 rs363227 (T allele) predicted investigator diagnosis (*P* = 0.02 and 0.05). However, none of the SNP findings were significant after correction for multiple comparisons.

**TABLE 4:** Dopamine‐related SNPs and cognitive impairment

Cognitive outcome	Genetic SNP								
	DRD3 rs6280 estimate (*P* value)	DRD5 rs6283 estimate (*P* value)	DRD5 rs1967550 estimate (*P* value)	SLC6A3 rs27072 estimate (*P* value)	DRD1 rs686 estimate (*P* value)	DDC rs1451375 estimate (*P* value)	SLC18A2 rs363387 estimate (*P* value)	SLC18A2 rs2015586 estimate (*P* value)	SLC18A2 rs363227 estimate (*P* value)
Cognitive impairment									
MoCA	0.081 (0.65)	−0.005 (0.98)	0.082 (0.67)	−0.006 (0.98)	−0.052 (0.78)	−0.071 (0.70)	−0.391 (0.20)	0.143 (0.46)	−0.067 (0.76)
Detailed cognitive testing	0.248 (0.21)	0.027 (0.89)	0.097 (0.64)	−0.062 (0.77)	0.098 (0.62)	−0.181 (0.36)	−0.303 (0.39)	0.030 (0.89)	−0.357 (0.13)
MDS‐UPDRS cognition score	0.243 (0.29)	0.333 (0.17)	0.222 (0.35)	0.030 (0.90)	0.439 (0.06)	0.017 (0.94)	−0.968 (0.01)	0.152 (0.55)	−0.139 (0.62)
Investigator diagnosis	0.095 (0.63)	0.043 (0.83)	0.124 (0.57)	−0.006 (0.98)	0.079 (0.70)	0.013 (0.95)	−0.260 (0.45)	0.307 (0.15)	0.212 (0.39)
Persistent cognitive impairment									
MoCA	−1.246 (0.02)	−0.004 (0.99)	0.310 (0.56)	−0.091 (0.87)	−0.959 (0.06)	−0.218 (0.68)	0.095 (0.91)	0.538 (0.33)	0.623 (0.29)
Detailed cognitive testing	0.089 (0.88)	0.654 (0.27)	0.312 (0.62)	−0.372 (0.56)	−0.246 (0.67)	0.569 (0.41)	0.455 (0.59)	1.139 (0.15)	0.440 (0.47)
MDS‐UPDRS cognition score	0.164 (0.73)	0.269 (0.58)	−0.642 (0.17)	0.706 (0.13)	0.829 (0.12)	0.135 (0.78)	0.032 (0.96)	0.847 (0.15)	0.064 (0.91)
Investigator diagnosis	0.691 (0.36)	1.346 (0.10)	0.004 (0.10)	−0.460 (0.56)	−0.746 (0.29)	−0.274 (0.68)	−0.381 (0.76)	−0.249 (0.73)	1.636 (0.05)

Cognitive outcome	Genetic SNP								
	SLC18A2 rs363276 estimate (*P* value)	DRD4 rs747302 estimate (*P* value)	DRD rs1800955 estimate (*P* value)	TH rs6356 estimate (*P* value)	DRD2 rs1800497 estimate (*P* value)	COMT rs4680 estimate (*P* value)	MAOB rs1799836 estimate (*P* value)	MAOB rs10521432 estimate (*P* value)	MAOB rs5905512 estimate (*P* value)
Cognitive impairment									
MoCA	0.117 (0.57)	−0.291 (0.11)	−0.127 (0.51)	−0.030 (0.87)	0.250 (0.16)	0.164 (0.46)	−0.202 (0.27)	−0.202 (0.33)	0.399 (0.03)
Detailed cognitive testing	0.076 (0.73)	0.016 (0.94)	0.093 (0.67)	0.265 (0.18)	0.201 (0.31)	0.133 (0.57)	0.037 (0.85)	0.052 (0.81)	0.246 (0.22)
MDS‐UPDRS cognition score	0.014 (0.96)	0.118 (0.62)	0.280 (0.29)	0.051 (0.82)	0.036 (0.87)	0.075 (0.77)	0.138 (0.55)	0.132 (0.59)	0.062 (0.77)
Investigator diagnosis	0.351 (0.12)	−0.201 (0.34)	−0.078 (0.71)	−0.114 (0.57)	0.034 (0.87)	0.155 (0.52)	−0.197 (0.31)	−0.317 (0.16)	0.367 (0.07)
Persistent cognitive impairment									
MoCA	0.169 (0.77)	−0.617 (0.26)	0.499 (0.38)	0.003 (0.99)	0.519 (0.32)	0.238 (0.66)	−0.524 (0.29)	−0.493 (0.33)	0.532 (0.29)
Detailed cognitive testing	0.500 (0.39)	0.328 (0.59)	0.375 (0.56)	0.347 (0.56)	0.359 (0.54)	0.100 (0.88)	−0.582 (0.32)	−0.580 (0.40)	0.610 (0.30)
MDS‐UPDRS cognition score	−0.006 (0.99)	0.334 (0.53)	1.287 (0.05)	−0.174 (0.71)	−0.094 (0.85)	0.754 (0.21)	−0.397 (0.42)	−0.770 (0.20)	0.178 (0.71)
Investigator diagnosis	1.769 (0.02)	1.241 (0.18)	−0.277 (0.68)	0.218 (0.76)	0.415 (0.55)	0.349 (0.68)	0.036 (0.96)	0.658 (0.35)	−0.299 (0.66)

#### LEDD Models

Higher total LEDD over time was associated with CI based on detailed cognitive testing (*P* = 0.01) and MDS‐UPDRS cognition score (*P* < 0.001) (Table 5). For persistent CI, higher LEDD over time was associated with all four cognitive outcomes: worse MoCA score (*P* = 0.002), detailed cognitive testing (*P* = 0.01), MDS‐UPDRS cognition score (*P* < 0.001), and investigator diagnosis (*P* = 0.003). Examining by most commonly‐used PD medications, the statistically‐significant effects seen for total LEDD were driven by levodopa LEDD rather than dopamine agonist LEDD (Table 5).

**TABLE 5:** Dopaminergic therapy and cognitive impairment

Cognitive impairment outcome	Change in total LEDD over time estimate (*P* value)	Change in levodopa LEDD over time estimate (*P* value)	Change in dopamine agonist LEDD over time estimate (*P* value)
Cognitive impairment			
MoCA	0.183 (0.20)	0.159 (0.27)	0.668 (0.33)
Detailed cognitive testing	0.408 (0.01)	0.491 (0.002)	−0.545 (0.46)
MDS‐UPDRS cognition score	0.629 (<0.001)	0.694 (<0.001)	−1.730 (0.10)
Investigator diagnosis	0.280 (0.18)	0.394 (0.03)	−1.767 (0.09)
Persistent cognitive impairment			
MoCA	1.607 (0.002)	1.475 (0.002)	0.562 (0.81)
Detailed cognitive testing	1.202 (0.01)	1.001 (0.02)	0.424 (0.87)
MDS‐UPDRS cognition score	1.183 (<0.001)	1.046 (0.002)	−0.005 (0.99)
Investigator diagnosis	2.266 (0.003)	2.669 (0.002)	−6.863 (0.11)

Abbreviation: LEDD: levodopa equivalent daily dose.

To determine if lower DAT SBR or worse motor symptoms are associated with higher LEDD, we then examined the association between time‐varying DAT binding and time‐varying LEDD, and found that lower mean striatum (*P* < 0.001), lowest putamen (*P* < 0.001) and mean caudate (*P* < 0.001) DAT binding predicted higher LEDD over time. There was no association between “on” MDS‐UPDRS motor score and LEDD over time (*P* = 0.137). We then reran all LEDD models with baseline mean striatum SBR entered as a covariate, and found that all significant p values for higher LEDD over time adversely impacting cognition remained significant when controlling for baseline DaTscan mean striatal SBR value.

## Discussion

The findings from this exploratory study provide preliminary evidence that the dopamine system is involved with cognitive decline in PD, including persistent CI. The measures predictive for impairment were nigrostriatal dopaminergic integrity and magnitude of chronic DRT; dopamine system‐related genes were not significant after correction for multiple comparisons. Other expected demographic and non‐motor clinical variables predicted long‐term decline (eg, increasing age, male sex, lower level of education, non‐White race, and increasing severity of anxiety and depression).

Lower baseline mean striatum DAT availability was associated with worse long‐term outcomes (detailed cognitive testing and MDS‐UPDRS cognition score). DaTscan (SPECT Ioflupane ^123^I) binds to the DAT, a molecule found pre‐synaptically on dopamine neurons in the nigrostriatal pathway. Longitudinal DaTscan results were not associated with cognitive outcomes, suggesting that it is early damage to the nigrostriatal, or downstream, dopamine pathways that contributes to decline. Mean striatal binding, not the most severely affected striatal region (ie, lowest putamen), predicted future decline. Analyses examining the caudate suggested that this region was driving the mean striatal binding results and suggests a key role for early dopaminergic cell loss in long‐term cognitive decline, consistent with the caudate being the striatal region most linked with cognitive abilities in PD,^43^ including in early disease.^18^, ^44^, ^45^ Finally, it is possible that some of the association between DAT binding and cognitive decline may be explained by comorbid psychiatric symptoms, as both depression and anxiety predicted worse cognitive course, and are known to be associated with DAT binding deficits.^46^, ^47^

There were consistent associations between higher amounts of DRT, assessed by calculating total LEDD longitudinally, and higher rates of CI. Examining the most commonly‐used PD medications that have a dosing range, the effect observed was driven by levodopa LEDD rather than dopamine agonist LEDD. While dopamine agonists are associated with development of psychosis and confusion in some patients, and chronic levodopa use is associated with non‐motor fluctuations including cognitive fluctuations, to our knowledge a direct association between higher LEDD, and specifically levodopa LEDD, over time and long‐term impairment has not been reported previously. The specific effect for levodopa might have been due to the more frequent use, and at higher doses, of levodopa; at the year 7 visit 94% of participants were on levodopa (mean levodopa LEDD = 274 mg/day), whereas only 45% were on a dopamine agonist (mean dopamine agonist LEDD = 63 mg/day).

As there was a significant correlation between the amount of DRT taken over time and severity of DAT deficit, it is possible that these two variables are assessing the same underlying dopamine‐related neurobiological deficit (ie, greater disease severity). However, adding baseline DaTscan mean striatum SBR value as a covariate to the LEDD models did not impact the results, suggesting that the adverse impact of higher dopaminergic therapy doses on cognition long‐term was not confounded by disease severity as measured by dopaminergic nigrostriatal dysfunction.

Six dopamine‐related SNPs were associated, but only before correction for multiple comparisons, with worse long‐term cognitive outcomes and may warrant further exploration. Three of them were VMAT2 SNPs, two non‐coding variants (rs363276 and rs363227) and one synonymous coding variant (rs363387), with two minor alleles (rs363276 and rs363227) and one major allele (rs363387) predicting worse cognition. VMAT2 is an integral presynaptic protein that regulates the packaging and subsequent release of dopamine and other monoamines from neuronal vesicles into the synapse, and also counteracts intracellular toxicity. Two SNPs were dopamine receptor genes, DRD3 and DRD4, both part of the D2‐like dopamine receptor subfamily associated with non‐motor features in PD. The minor allele of DRD3 (rs6280) associated with CI codes for a serine for glycine substitution. The minor allele of DRD4 (rs1800955) associated with impairment is a non‐coding variant. There is little evidence for impact of genetic polymorphisms in dopamine receptors on cognitive course in PD beyond a single, small study reporting an association between DRD2 and long‐term cognitive decline.^21^ Finally, the minor allele of one MAO‐B SNP (rs5905512), an intron variant, was associated with CI. MAO‐B is one of two MAO isoenzymes located on the X chromosome and metabolizes dopamine; while several MAO‐B inhibitors are approved for the treatment of motor symptoms, a randomized controlled trial of a selective MAO‐B inhibitor for the treatment of PD‐MCI was negative.^30^

We defined cognitive impairment four different ways, which increased the likelihood of an uncorrected, significant p value, but also complicates interpretation given varied results across neurobiological measures and cognitive impairment definitions. The most consistent effects were seen for^1^ detailed cognitive testing and^2^ MDS‐UPDRS Part I cognition score. What those two cognitive impairment assessment methods have in common, in comparison with MoCA cut‐off and site investigator diagnosis, is having the lowest frequency of diagnosed cognitive impairment at all‐time points starting with year 2 forward. This suggests that these ways of assessing cognitive impairment may have better specificity (ie, fewer false positives) and therefore more likely to detect a neurobiological signal.

A limitation of the research is our inability to examine, and control for, other biological predictors of cognitive decline in PD that might be associated with nigrostriatal dopamine system‐related biomarkers, including other dopamine pathways (eg, mesolimbic or mesocortical) and other neurotransmitter systems (eg, cholinergic and noradrenergic). For instance, if neuropathophysiological changes to the nigrostriatal DS are correlated with changes to the cortical cholinergic system^48^, ^49^ or to white matter hyperintensities,^50^ then DAT binding deficits may be a proxy for cholinergic or white matter dysfunction that might directly be leading to cognitive decline.^51^, ^52^, ^53^ However, in our study it was lower DAT availability at the time of diagnosis that predicted long‐term impairment, a stage at which cholinergic deficits are present but not pronounced.^54^ Other limitations were a priori determining which dopamine‐related SNPs to include, and not having access to other measures of the dopamine system that are associated with cognitive decline in PD in preliminary studies (eg, neurodegeneration in the substantia nigra, PET imaging of D2 receptors, and retinal thinning in dopamine layers).^55^, ^56^, ^57^ Although the use of a subgroup of individuals with incident and persistent CI was meant to reduce clinical heterogeneity, the number of participants reaching this endpoint was small. Due to attrition and COVID‐19, the original cohort decreased in size by 43% by year 7. Finally, this is an exploratory study and positive SNP results did not withstand correction for multiple comparisons, and even positive findings for DaTscan would not withstand a more stringent correction, so all positive findings require replication.

Dysfunction of the dopamine system is central to diagnosis and progression of PD, particularly its motor features. While the dopamine system, including dopaminergic medications, have been implicate in the etiology of certain psychiatric features in PD, little is known about its impact on long‐term cognitive course. These results, from a relatively large, longitudinal, biomarker‐rich cohort study with a range of cognitive assessments, suggest that the dopamine system in PD is implicated not only in acute, early or domain‐specific cognitive changes, but also in long‐term CI, an outcome of great clinical significance to patients.

## Author Roles

(1) Research project: A. Conception, B. Organization, C. Execution; (2) Statistical Analysis: A. Design, B. Execution, C. Review and Critique; (3) Manuscript Preparation: A. Writing of the first draft, B. Review and Critique

D.W.: 1A, 1C, 3A, 3B

M.P.: 3A, 3B

H.R.C.: 2A, 2B, 3A, 3B

C.C.G.: 2A, 2B, 3A, 3B

C.B.: 3A, 3B

E.B.: 3A, 3B

L.M.C.: 3A, 3B

C.S.C.: 3A, 3B

R.D.D.: 3A, 3B

T.F.: 3A, 3B

D.G.: 3A, 3B

K.K.: 3A, 3B

K.M.: 3A, 3B

K.M.: 3A, 3B

B.M.: 3A, 3B

K.L.P.: 3A, 3B

T.S.: 3A, 3B

A.S.: 3A, 3B

A.S.: 3A, 3B

J.S.: 3A, 3

C.M.T.: 3A, 3B

## Disclosures

**Ethical Compliance Statement:** An ethical standards committee on human experimentation reviewed and approved the study at each site. Additionally, the Western Institutional Review Board at the University of Florida reviewed this study (Protocol #20200597). At the University of Pennsylvania, the Institutional Review Board approved this research (Protocol #843441). All participant signed an approved informed consent form. We confirm that we have read the Journal's position on issues involved in ethical publication and affirm that this work is consistent with those guidelines.

**Funding Sources and Conflicts of Interest:** PPMI—a public‐private partnership—is funded by the Michael J. Fox Foundation for Parkinson's Research and funding partners, including 4D Pharma, AbbVie Inc., AcureX Therapeutics, Allergan, Amathus Therapeutics, Aligning Science Across Parkinson's (ASAP), Avid Radiopharmaceuticals, Bial Biotech, Biogen, BioLegend, Bristol Myers Squibb, Calico Life Sciences LLC, Celgene Corporation, DaCapo Brainscience, Denali Therapeutics, The Edmond J. Safra Foundation, Eli Lilly and Company, GE Healthcare, GlaxoSmithKline, Golub Capital, Handl Therapeutics, Insitro, Janssen Pharmaceuticals, Lundbeck, Merck & Co., Inc., Meso Scale Diagnostics, LLC, Neurocrine Biosciences, Pfizer Inc., Piramal Imaging, Prevail Therapeutics, F. Hoffmann‐La Roche Ltd and its affiliated company Genentech Inc., Sanofi Genzyme, Servier, Takeda Pharmaceutical Company, Teva Neuroscience, Inc., UCB, Vanqua Bio, Verily Life Sciences, Voyager Therapeutics, Inc. and Yumanity Therapeutics, Inc. (www.ppmi-info.org/about-ppmi/who-we-are/study-sponsors).

**Financial Disclosures for the Previous 12 Months:** In the past year Dr. Weintraub has received research funding or support from Michael J. Fox Foundation for Parkinson's Research, Alzheimer's Therapeutic Research Initiative (ATRI), Alzheimer's Disease Cooperative Study (ADCS), International Parkinson and Movement Disorder Society (IPMDS), National Institute on Health (NIH), Parkinson's Foundation; U.S. Department of Veterans Affairs and Acadia Pharmaceuticals; honoraria for consultancy from Acadia Pharmaceuticals, Alkahest, Aptinyx, Cerevel Therapeutics, CHDI Foundation, Clintrex LLC (Otsuka), EcoR1 Capital, Eisai, Ferring, Gray Matter Technologies, Great Lake Neurotechnologies, Intra‐Cellular Therapies, Janssen, Merck, Sage, Scion and Signant Health; and license fee payments from the University of Pennsylvania for the QUIP and QUIP‐RS. No other authors have a financial relationship related to content of manuscript, and will provide full disclosures on request. The authors report no conflicts of interest in the previous 12 months.

## Supporting information


**TABLE S1.** SNP alleles and frequency

## Data Availability

Data used in the preparation of this article were obtained (February 1, 2021) from the Parkinson's Progression Markers Initiative (PPMI), RRID:SCR_006431. For up‐to‐date information on the study, visit www.ppmi-info.org.

## Appendix

(*and the Parkinson's Progression Markers Initiative)

Ruth Schneider, MD (University of Rochester, Rochester, NY); Kelvin Chou, MD (University of Michigan, Ann Arbor, MI); David Russell, MD, PhD (Institute for Neurodegenerative Disorders, New Haven, CT); Stewart Factor, DO (Emory University of Medicine, Atlanta, GA); Penelope Hogarth, MD (Oregon Health and Science University, Portland, OR); Robert Hauser, MD, MBA (University of South Florida, Tampa, FL); Nabila Dahodwala, MD, MSc (University of Pennsylvania, Philadelphia, PA); Marie H Saint‐Hilaire, MD, FRCPC, FAAN (Boston University, Boston, MA); David Shprecher, DO (Banner Research Institute, Sun City, AZ); Hubert Fernandez, MD (Cleveland Clinic, Cleveland, OH); Kathrin Brockmann, MD (University of Tuebingen, Tuebingen, Germany); Yen Tai, MD, PhD (Imperial College of London, London, UK); Paolo Barone, MD, PhD (University of Salerno, Salerno, Italy); Stuart Isaacson, MD (Parkinson's Disease and Movement Disorders Center, Boca Raton, FL); Alberto Espay, MD, MSc, FAAN, FANA (University of Cincinnati, Cincinnati, OH); Maria Jose Martí, MD, PhD (Hospital Clinic of Barcelona, Barcelona, Spain); Eduardo Tolosa MD, PhD (Hospital Clinic of Barcelona, Barcelona, Spain); Shu‐Ching Hu, MD, PhD (University of Washington, Seattle, WA); Emile Moukheiber, MD (Johns Hopkins University, Baltimore, MD); Jean‐Christophe Corvol, MD (University Hospitals Pitié Salpêtrière, Paris, France); Nir Giladi, MD (Tel Aviv Sourasky Medical Center, Tel Aviv, Israel); Javier Ruiz Martinez, MD, PhD (Hospital Universitario Donostia, San Sebastian, Spain); Jan O. Aasly, MD (St. Olav's University Hospital, Trondheim, Norway); Leonidas Stefanis, MD, PhD (National and Kapodistrian University of Athens, Athens, Greece); Karen Marder, MD MPH (Columbia University Irving Medical Center, New York, NY); Arjun Tarakad, MD (Baylor College of Medicine, Houston, TX); Connie Marras, MD, PhD, FRCP(C) (Toronto Western Hospital, Toronto, Canada); Tiago Mestre, MD, PhD (The Ottawa Hospital, Ottawa, Canada); Aleksandar Videnovic, MD, MSc (Massachusetts General Hospital, Boston, MA); Rajesh Pahwa, MD (University of Kansas Medical Center, Kansas City, KS); Mark Lew, MD (University of Southern California, Los Angeles, CA); Holly Shill, MD (Barrow Neurological Institute, Phoenix, AZ); Amy Amara, MD, PhD (University of Alabama at Birmingham, Birmingham, AL); Charles Adler, MD, PhD (Mayo Clinic Arizona, Scottsdale, AZ); Susan Bressman, MD (Mount Sinai Beth Israel, New York, NY); Maureen Leehey, MD (University of Colorado, Aurora, CO); Giulietta Riboldi, MD (NYU Langone Medical Center, New York, NY); Nikolaus McFarland, MD, PhD, FAAN (University of Florida, Gainesville, FL); Ron Postuma, MD, FRCPC (Montreal Neurological Institute and Hospital/McGill, Montreal, QC, Canada); Werner Poewe, MD (Innsbruck Medical University, Innsbruck, Austria); Zoltan Mari, MD (Cleveland Clinic‐Las Vegas Lou Ruvo Center for Brain Health, Las Vegas, NV); Nicola Pavese, MD, PhD (Clinical Ageing Research Unit, Newcastle, UK); Michele Hu, MD, PhD (John Radcliffe Hospital Oxford and Oxford University, Oxford, UK); Norbert Brüggemann, MD (Universität Lübeck, Luebeck, Germany); Christine Klein, MD, FEAN (Universität Lübeck, Luebeck, Germany); Bastiaan Bloem, MD, PhD (Radboud University, Nijmegen, Netherlands).
