# Supplementary material for: Impact of the Dopamine System on Long‐Term Cognitive Impairment in Parkinson Disease: An Exploratory Study
Source: Mov Disord Clin Pract. 2023 Apr 25;10(6):943–55. doi: 10.1002/mdc3.13751 (PMC10272925; doi:10.1002/mdc3.13751)
Supplement: Supplementary file 1 — TABLE S1. SNP alleles and frequency [file MDC3-10-943-s001.docx]

**Supplementary Table 1. SNP alleles and frequency**

| **Single nucleotide polymorphisms (SNPs)** | **Allele 1** | **Allele 2** | **Allele 2 frequency*** |
| --- | --- | --- | --- |
| DRD1 rs686 | A | G | 61.69 |
| DRD2 Taq 1A rs1800497 | G | A | 37.81 |
| DRD3 rs6280 | T | C | 50.50 |
| DRD4 rs747302 | C | G | 66.92 |
| DRD4 rs1800955 | T | C | 68.91 |
| DRD5 rs6283 | T | C | 56.72 |
| DRD5 rs1967550 | T | G | 67.91 |
| SLC6A3_rs27072 | C | T | 31.09 |
| DDC rs1451375 | C | A | 59.95 |
| SLC18A2 rs363387 | T | G | 9.20 |
| SLC18A2 rs2015586 | T | C | 68.16 |
| SLC18A2 rs363227 | C | T | 21.14 |
| SLC18A2 rs363276 | C | T | 28.11 |
| TH rs6356 | C | T | 59.95 |
| COMT rs4680 | A | G | 71.64 |
| MAOB rs1799836 | T | C | 56.21 |
| MAOB rs10521432 | G | A | 33.58 |
| MAOB rs5905512 | G | A | 57.21 |

*Frequency is the percentage of participants with at least one Allele 2.

DRD: dopamine receptor D

SLC: solute carriers

DDC: dopamine decarboxylase

TH: tyrosine hydroxylase

COMT: catechol-O-methyltransferase

MAOB: monoamine oxidase-B
